# Persistent Alzheimer-related molecular dysregulation after cerebral ischemia: a hierarchical-circular hypothesis of impaired allostatic recovery

**DOI:** 10.3389/fnagi.2026.1938670

**Published:** 2026-09-29

**Authors:** Samuel Ruesga Mundo

**Affiliations:** High Specialty Medical Unit, Western National Medical Center, Mexican Institute of Social Security (IMSS), Guadalajara, Mexico

**Keywords:** allostatic integrity, Alzheimer’s disease, biological memory, brain ischemia, epigenetic memory, gene expression, hierarchical-circular model, neuroinflammation

## Abstract

**Introduction:**

Growing evidence suggests a consistent association between brain ischemia and the subsequent development of Alzheimer’s disease (AD)-related molecular changes. Experimental and clinical studies have demonstrated that ischemic episodes trigger the overexpression of genes associated with AD pathology, including amyloid precursor protein (APP), β-secretase (BACE1), presenilin 1 and 2 (PSEN1, PSEN2), and MAPT (tau). However, the mechanisms by which transient or chronic ischemic events convert transient gene dysregulation into sustained neurodegenerative pathology remain poorly understood. We acknowledge that current evidence, largely derived from animal models, does not establish that ischemia alone is sufficient to initiate AD as defined by the 2024 NIA-AA biological criteria (core amyloid and tau biomarkers); rather, ischemia may act as a significant contributor that accelerates or exacerbates AD-related molecular changes in vulnerable individuals.

**Objective:**

To propose a multilevel reconceptualization of the ischemic contribution to AD within the hierarchical-circular model of biological memory and the construct of allostatic integrity, integrating recent evidence on post-ischemic gene expression, neuroinflammation, oxidative stress, and epigenetic mechanisms.

**Hypothesis and synthesis:**

We propose that cerebral ischemia acts as a high-intensity allostatic challenge that may “freeze” biological memory into a pathological configuration. Within this framework, ischemic episodes are not merely transient vascular events but rather serve as biological “programmers” that dysregulate AD-associated genes across multiple levels of biological organization: genetic-epigenetic (DNA methylation, microRNA dysregulation), cellular-mitochondrial (oxidative stress, mitophagy failure), synaptic-neurotrophic (loss of plasticity), neuroimmune-proteostatic (chronic neuroinflammation, protein misfolding), and behavioral-cognitive (progressive dementia). The persistent overexpression of APP, BACE1, PSEN1, PSEN2, and MAPT represents a failure of the system’s capacity to restore adaptive circular information flow after an ischemic insult.

**Implications:**

This model reframes the ischemic contribution to AD from a linear cause-effect relationship to a dynamic, multilevel failure of allostatic adaptation. It provides a conceptual roadmap for understanding why ischemic events may predispose to sporadic AD and suggests that therapeutic strategies should target the restoration of allostatic integrity across multiple levels rather than single molecular pathways.

**Testable predictions:**

The framework generates falsifiable predictions, including: (1) composite indices of allostatic integrity will predict post-ischemic AD progression better than single biomarkers; (2) interventions that restore allostatic integrity (e.g., anti-inflammatory agents, epigenetic modulators, neuroprotective strategies) will attenuate AD-associated gene dysregulation; and (3) patients with higher allostatic load prior to an ischemic event will show more severe AD-associated gene expression changes and worse clinical outcomes.

## 1 Introduction

Alzheimer’s disease (AD) and related dementias represent one of the greatest public health challenges of the 21st century, with global prevalence expected to rise sharply in the coming decades (GBD 2019 Dementia Forecasting Collaborators, 2022). Despite unprecedented advances in neuroimaging, biomarkers, and genetics, disease-modifying therapies have produced only modest clinical benefits, and most pharmacological trials continue to fail (Cummings et al., 2024). This therapeutic impasse has fueled growing recognition that predominant linear models—centered on beta-amyloid, tau, and single-pathway cascades–are insufficient to capture the full complexity of dementia pathogenesis (Long and Holtzman, 2019).

Among the most compelling emerging hypotheses is the contribution of brain ischemia to AD pathology. Increasing evidence from experimental and clinical studies demonstrates that brain ischemia–whether from cardiac arrest, stroke, or chronic cerebrovascular disease—triggers the overexpression of genes intimately associated with AD pathology. These include amyloid precursor protein (APP), β-secretase (BACE1), presenilin 1 and 2 (PSEN1, PSEN2), and MAPT (tau) (Pluta and Ułamek-Kozioł, 2019; Pluta and Ulamek-Koziol, 2024). In the CA1 and CA3 regions of the hippocampus and temporal cortex–areas critically involved in memory formation and selectively vulnerable to both ischemia and AD–these genes are significantly deregulated following ischemic episodes (Pluta, 2022).

The persistence of this gene dysregulation is particularly striking. Studies in animal models have demonstrated that presenilin 1 and 2 gene expression remains significantly elevated at all time points following ischemia, and MAPT expression remains elevated throughout the observation period, with peak expression occurring as late as 12 months post-ischemia (Pluta et al., 2020). Similarly, apolipoprotein genes associated with AD risk show sustained alterations in the frontal cortex for up to 2 years after an ischemic episode (Pluta et al., 2025). These findings suggest that ischemia does not merely cause transient changes but rather may program a sustained pathological gene expression profile that can persist for years.

Despite this growing body of evidence, the mechanisms by which ischemic events convert transient gene dysregulation into sustained neurodegenerative pathology remain incompletely understood. Current models tend to focus on isolated molecular pathways–amyloidogenesis, tau phosphorylation, neuroinflammation, or oxidative stress–without providing an integrated framework that explains how these processes interact across time and levels of biological organization (Serrano-Pozo et al., 2024). Importantly, we acknowledge that much of the direct evidence for post-ischemic gene dysregulation comes from animal models, and translation to human disease requires careful validation. Furthermore, the 2024 NIA-AA biological criteria define Alzheimer’s disease by core biomarkers (amyloid and tau) rather than by cognitive symptoms or ischemic changes alone (Jack et al., 2018, 2024). Therefore, while ischemia may contribute to or accelerate AD-related molecular pathology, it should not be equated with a direct “ischemic etiology” of AD as biologically defined.

Recent conceptual and diagnostic advances have deepened, rather than resolved, the paradox of dissociation between pathology and clinical expression. The 2024 NIA-AA criteria have clarified the biological continuum of disease but have also highlighted that individuals with substantial amyloid and tau pathology may remain cognitively intact, whereas others with relatively modest biomarker changes exhibit rapid decline (Savva et al., 2009). These discrepancies suggest that dementia cannot be fully understood as a simple downstream consequence of aggregated proteins, but instead reflects broader disturbances in how biological systems encode, integrate, and stabilize information over time.

Converging evidence points to the importance of lifespan, multisystem influences that are only partially addressed by current frameworks (Livingston et al., 2020). Large longitudinal cohorts now show that elevated allostatic load–a composite index of cumulative physiological “wear and tear” across metabolic, cardiovascular, and inflammatory systems–is prospectively associated with increased risk for all-cause dementia and specific subtypes, including Alzheimer’s disease and vascular dementia (Gou et al., 2025; Smith et al., 2025; Buller-Peralta et al., 2025). These findings support the view that chronic stress physiology functions as a key mediator between environmental exposures and late-life cognitive decline (Souza-Talarico et al., 2025). At the same time, emerging studies link adverse experiences across the life-course, including childhood abuse and adult economic hardship, to higher dementia risk (Livingston et al., 2020), underscoring the role of social and psychological stressors in shaping long-term vulnerability.

At the molecular level, gene-environment interactions and epigenetic mechanisms provide a plausible route by which early and chronic stress exposures become biologically embedded (Nothling et al., 2025; Yusupov et al., 2024). Work on stress-responsive genes such as FKBP5 and risk alleles like APOE-ε4 demonstrates that specific genetic variants modulate epigenetic plasticity, hypothalamic-pituitary-adrenal (HPA) axis reactivity, and neurophysiological activity patterns in aging populations (Zannas et al., 2016; Verduzco Espinoza et al., 2025). These data reinforce the idea that genetic architecture and epigenetic “memory” jointly calibrate stress responses and neural network function across the lifespan, thereby influencing who will convert from mild cognitive impairment to dementia under comparable neuropathological conditions (Panitch et al., 2024; Klengel et al., 2013).

In this article, we apply the hierarchical-circular model of biological memory–recently described in detail elsewhere (Ruesga Mundo, 2026)–specifically to the context of cerebral ischemia. We argue that cerebral ischemia may act as a high-intensity allostatic challenge that can “freeze” biological memory into a pathological configuration, perpetuating AD-associated gene dysregulation across multiple levels of biological organization. This framework integrates recent evidence on post-ischemic gene expression, neuroinflammation, oxidative stress, epigenetic mechanisms, and protein misfolding into a unified multilevel hypothesis.

The objective of this article is to synthesize emerging evidence on the genomic and proteomic consequences of brain ischemia within the hierarchical-circular model, focusing on how ischemia may impair allostatic recovery and contribute to sustained AD-related molecular dysregulation. We aim to develop an integrative framework that conceptualizes the ischemic contribution to AD as a failure of allostatic integrity, generates testable predictions for biomarker development and therapeutic research, and provides a conceptual roadmap for future investigations in this rapidly evolving field. The hierarchical-circular organization of the proposed framework is summarized in Figure 1.

**FIGURE 1:**
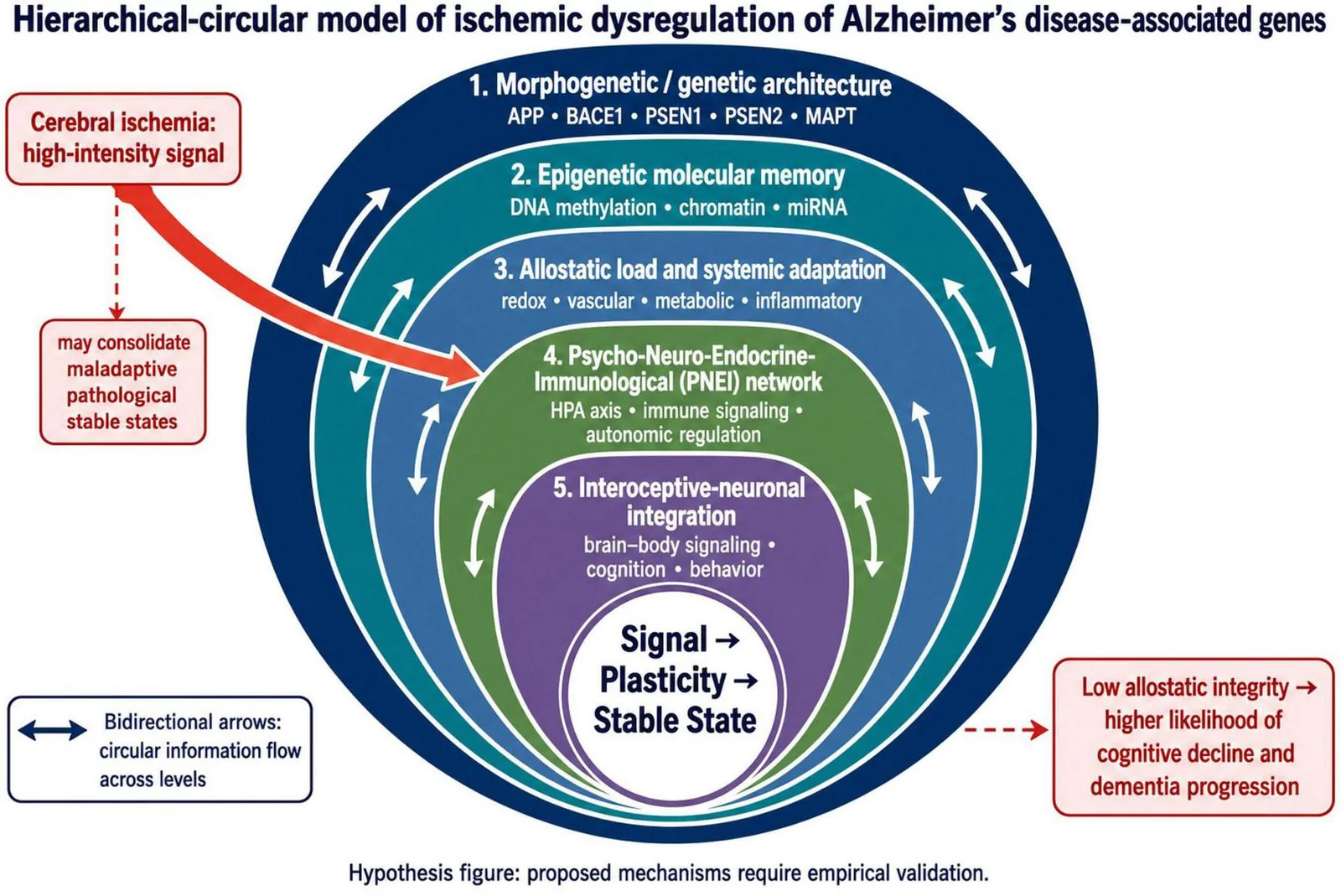
Hierarchical-circular model of ischemic dysregulation of Alzheimer’s disease-associated genes. The model depicts five interconnected levels of biological memory–(1) morphogenetic/genetic architecture, (2) epigenetic molecular memory, (3) allostatic load and systemic adaptation, (4) the Psycho-Neuro-Endocrine-Immunological (PNEI) network, and (5) interoceptive-neuronal integration—organized around the unifying principle “Signal→Plasticity→Stable State.” Bidirectional arrows indicate circular information flow, whereby perturbations at any level can propagate through the system and consolidate maladaptive pathological stable states. Cerebral ischemia acts as a high-intensity signal that may “freeze” biological memory into a pathological configuration, perpetuating AD-associated gene dysregulation (APP, BACE1, PSEN1, PSEN2, MAPT). Low allostatic integrity increases the likelihood of progression to dementia.

## 2 The ischemic contribution to Alzheimer’s disease: evidence for gene dysregulation

### 2.1 Post-ischemic overexpression of AD-associated genes

A substantial body of evidence supports the role of brain ischemia in triggering and sustaining the expression of genes central to AD pathology. Experimental studies using animal models of transient global cerebral ischemia have consistently demonstrated that ischemic episodes induce the overexpression of:

- Amyloid precursor protein (APP): Ischemia increases the amyloidogenic processing of APP, leading to the accumulation of Aβ peptides in the brain (Pluta et al., 2020). Hypoxia and ischemia also trigger endoplasmic reticulum stress and impair autophagy, which can stimulate the expression of presenilin 1 and activate γ-secretase (Pluta and Ulamek-Koziol, 2024).
- β-secretase (BACE1): The gene encoding the rate-limiting enzyme for Aβ production is significantly upregulated following ischemic injury (Pluta and Ułamek-Kozioł, 2019).
- Presenilin 1 and 2 (PSEN1, PSEN2): These genes, which encode components of the γ-secretase complex, show significantly elevated expression at all time points following ischemia (Pluta et al., 2020; Pluta, 2022).
- MAPT (tau): MAPT expression is significantly elevated throughout the post-ischemic observation period, with peak expression occurring 12 months after the ischemic episode (Pluta et al., 2020).
- Apolipoproteins: Genes encoding apolipoproteins A1, E, and J show sustained alterations in the frontal cortex following ischemia, with changes persisting for up to 2 years (Pluta et al., 2025).
- LRP1 and RAGE: These receptors, involved in Aβ clearance and transport across the blood-brain barrier, show altered expression in the frontal cortex in ischemic models of AD (Pluta et al., 2025).
- α-Synuclein: This protein, implicated in both AD and synucleinopathies, shows altered gene expression in the hippocampal CA3 subfield following ischemia (Pluta et al., 2025).

The pattern of dysregulation is region-specific, with the CA1 and CA3 regions of the hippocampus and the temporal cortex showing the most consistent changes (Pluta, 2022). Notably, these regions are among the most selectively vulnerable to both ischemia and AD pathology, suggesting a shared vulnerability that may underlie the ischemic contribution to sporadic AD.

### 2.2 The temporal dynamics of post-ischemic gene expression

The temporal dynamics of post-ischemic gene expression are particularly revealing. Studies tracking gene expression for up to 2 years following a single ischemic episode have demonstrated that dysregulation is not a transient phenomenon but rather a sustained pathological reprogramming (Pluta et al., 2020, 2025).

For example, presenilin 1 and 2 gene expression remains significantly elevated at 2, 7, and 30 days, as well as at 6, 12, 18, and 24 months post-ischemia (Pluta et al., 2020). MAPT expression follows a similar pattern, with peak expression occurring at 12 months (Pluta et al., 2020). Apolipoprotein gene alterations persist for the entire two-year observation period (Pluta et al., 2025).

This sustained dysregulation suggests that ischemia does not merely cause a transient stress response but rather may program a persistent pathological gene expression profile. The fact that gene expression changes can be detected years after a single ischemic episode implies the existence of mechanisms that maintain and perpetuate the pathological state–mechanisms that we propose are rooted in failures of allostatic integrity and the “freezing” of biological memory. The proposed hierarchical-circular model of ischemic dysregulation of Alzheimer’s disease-associated genes is summarized in Figure 1.

## 3 The hierarchical-circular model of biological memory: application to ischemia

### 3.1 Foundational concepts

The hierarchical-circular model of biological memory has been described in detail elsewhere (Ruesga Mundo, 2026). Briefly, the model proposes that organisms maintain adaptive function through the continuous, circular flow of information across five interconnected levels of biological organization:

- Morphogenetic programming and genetic architecture: Constitutive genetic variants that establish baseline vulnerabilities and resilience capacities.
- Epigenetic molecular memory: DNA methylation, histone modifications, and non-coding RNA regulation that encode past experiences.
- Allostatic load and systemic physiological adaptation: Cumulative physiological “wear and tear” across metabolic, cardiovascular, and inflammatory systems.
- The Psycho-Neuro-Endocrine-Immunological (PNEI) network: Bidirectional communication pathways linking psychological states, brain function, peripheral physiology, and immune responses.
- Interoceptive-neuronal integration: The integration of internal bodily signals with neuronal dynamics across large-scale brain networks.

Within this framework, health represents a state of “allostatic integrity”—the capacity of the organism to maintain adaptive circular information flow across multiple levels (Ruesga Mundo, 2026). Disease, conversely, represents a failure of allostatic integrity—a “freezing” of biological memory in which pathological feedback loops become self-perpetuating and the system loses its capacity for adaptive reorganization.

The model is organized around the unifying principle “Signal→Plasticity→Stable State.” Biological systems continuously receive signals from their internal and external environments, undergo plastic changes in response to these signals, and subsequently settle into stable states that encode the history of prior signals. This triadic dynamic operates iteratively, such that each stable state becomes the basis for responding to subsequent signals, establishing circular causality across time (McNamara et al., 2024; Barrett and Simmons, 2015).

Allostatic load–the cumulative physiological wear and tear resulting from chronic stress (McEwen and Stellar, 1993)–plays a central role in this framework. Prolonged exposure to stress mediators progressively damages brain structures associated with cognitive functions, increasing the risk of AD (Guidi et al., 2021). Higher allostatic load has been associated with increased brain amyloid accumulation, suggesting that allostatic load may play a role in AD pathophysiology (Smith et al., 2025).

### 3.2 Ischemia as a high-intensity allostatic challenge

Within this framework, cerebral ischemia can be conceptualized as a high-intensity allostatic challenge that may overwhelm the system’s adaptive capacity. The acute metabolic crisis induced by ischemia—oxygen and glucose deprivation, ATP depletion, excitotoxicity, and oxidative stress—represents a severe perturbation of cellular-mitochondrial homeostasis (Level 2) that cascades across all other levels.

Critically, we propose that ischemia does not merely cause transient damage but may “freeze” biological memory into a pathological configuration. The sustained overexpression of AD-associated genes–APP, BACE1, PSEN1, PSEN2, MAPT, and apolipoproteins–represents a failure of the system to restore adaptive circular information flow after the ischemic insult. Instead of returning to baseline, gene expression may become locked in a maladaptive state that perpetuates neurodegeneration.

This “freezing” may be maintained through pathological feedback loops across multiple levels:

- Genetic-epigenetic level: Ischemia-induced changes in DNA methylation and microRNA expression may silence protective genes and activate pathological genes (Pluta and Ułamek-Kozioł, 2019; Yusupov et al., 2024).
- Cellular-mitochondrial level: Persistent oxidative stress and mitochondrial dysfunction create a bioenergetic crisis that sustains pathology (Pluta and Ulamek-Koziol, 2024).
- Synaptic-neurotrophic level: Reduced neurotrophic support and loss of synaptic plasticity impair cognitive function.
- Neuroimmune-proteostatic level: Chronic neuroinflammation and protein misfolding create a self-perpetuating cycle of damage (Pluta et al., 2025; Feng et al., 2025).
- Behavioral-interoceptive level: Progressive cognitive decline and loss of functional independence further increase allostatic load (Hazelton et al., 2025a,b).

### 3.3 Hypothesized mechanisms of circular propagation in post-ischemic pathology

To move beyond abstract circularity, we propose concrete, testable mechanisms of bidirectional propagation. We emphasize that these pathways remain hypothetical and require direct empirical validation in human post-ischemic cohorts:

- Peripheral inflammation→epigenetic modification→HPA dysregulation (bidirectional). We hypothesize that peripheral IL-6 may cross the blood-brain barrier, activate NF-κB in microglia, and induce DNMT1-mediated hypermethylation of the NR3C1 (glucocorticoid receptor) promoter, reducing cortisol sensitivity and increasing allostatic load. Support for this general mechanism comes from studies on stress-related epigenetic modifications (Tyrka et al., 2016; Klengel et al., 2013; Zannas et al., 2016). In the ischemic context, this pathway may explain how post-ischemic inflammation becomes chronically embedded through epigenetic mechanisms; however, direct testing in ischemic populations is needed.
- Neuroimmune ↔ HPA axis (Levels 2 ↔ 4). Ischemic injury activates microglia and astrocytes, releasing pro-inflammatory cytokines that may induce DNA methylation changes at stress-related loci (FKBP5, NR3C1), thereby altering HPA axis reactivity and promoting sustained neuroinflammation. This bidirectional interaction is supported by evidence that peripheral inflammation can precede central pathology by years (Kim et al., 2025; Liu et al., 2023), but the specific post-ischemic chain remains a hypothesis of our model.
- Allostatic load→neuroinflammation→interoceptive network disruption (Levels 3→4→5). Elevated allostatic load may promote peripheral cytokine production, activate brain endothelial cells, upregulate adhesion molecules, and facilitate monocyte trafficking into the salience network (including anterior insula and anterior cingulate), impairing interoceptive prediction accuracy and further increasing allostatic load through poor self-regulation (Hazelton et al., 2025a,b).

These hypothesized pathways provide empirically testable causal links and are informed by direct evidence from non-ischemic human cohorts; their specific application to post-ischemic AD represents a key prediction of our framework.

## 4 Multilevel synthesis: ischemia-induced dysregulation of AD-associated genes

### 4.1 Level 1: morphogenetic programming and genetic architecture

The first level encompasses the genetic blueprint that establishes baseline vulnerabilities and resilience capacities across the lifespan. This includes constitutive genetic variants—such as APOE ε2/ε3/ε4 alleles–that modulate lipid metabolism, neuroinflammation, and synaptic integrity (Verduzco Espinoza et al., 2025). However, genetic architecture is not merely deterministic; it operates through dynamic interactions with environmental signals, a principle encapsulated in the concept of differential susceptibility (Belsky and Pluess, 2009).

In the context of ischemic AD, genetic variants may influence susceptibility to post-ischemic gene dysregulation. Verduzco Espinoza et al. (2025) demonstrated that APOE4 influences neuronal lipid metabolism and network activity through microglia-to-neuron signaling, revealing a mechanistic pathway that is modulated by inflammatory states. This finding suggests that the impact of APOE4 on post-ischemic gene expression may depend on the inflammatory environment–an environmental factor that is itself shaped by ischemia.

Morphogenetic programming refers to the processes by which genetic information guides tissue organization, neural circuit formation, and organ system development during critical windows. Early-life signals–including nutritional status, stress exposure, and maternal care–can calibrate these developmental trajectories, establishing set points that persist into adulthood (Danese and McEwen, 2012). This has profound implications for ischemic AD: individuals who experienced adverse early-life conditions may have a lower threshold for ischemia-induced gene dysregulation.

Within the model, Level 1 provides the foundational architecture upon which higher-level processes operate. However, consistent with circular dynamics, information from other levels, particularly epigenetic modifications (Level 2) and allostatic load (Level 3), can feed back to influence gene expression patterns, effectively updating the functional consequences of the genetic blueprint across the lifespan.

### 4.2 Level 2: epigenetic molecular memory

The second level captures the molecular mechanisms through which environmental signals–including ischemic insults–become biologically inscribed and maintained over time. Epigenetic modifications–including DNA methylation, histone acetylation, and non-coding RNA regulation–constitute a form of molecular memory that encodes past experiences and shapes future cellular responses (Zannas et al., 2016; Yusupov et al., 2024).

Work on stress-responsive genes has been particularly illuminating. Klengel et al. (2013) demonstrated that childhood trauma induces allele-specific DNA demethylation of FKBP5, a key regulator of glucocorticoid receptor sensitivity, leading to long-term alterations in HPA axis reactivity. Nothling et al. (2025) extended these findings by showing that FKBP5 methylation patterns mediate the relationship between childhood trauma and stress-related psychopathology. Similarly, Panitch et al. (2024) identified APOE genotype-specific methylation patterns linked to Alzheimer’s disease pathology and estrogen response, illustrating how genetic architecture (Level 1) and epigenetic memory (Level 2) interact to shape disease risk.

In the ischemic context, ischemia induces sustained changes in DNA methylation patterns that can persist for extended periods. These changes may silence protective genes while activating pathological genes such as APP, BACE1, and PSEN1 (Pluta and Ułamek-Kozioł, 2019, 2020). Non-coding RNAs play a direct and indirect role in promoting neuroinflammation in the post-ischemic brain (Pluta et al., 2025). Ischemia-induced changes in microRNA expression may perpetuate AD-associated gene dysregulation and contribute to sustained neurodegeneration.

Yusupov et al. (2024) investigated DNA methylation patterns of FKBP5 regulatory regions in both brain and blood of humanized mice and humans, demonstrating concordance between central and peripheral tissues. This finding is critical because it suggests that peripheral epigenetic markers may serve as accessible proxies for brain epigenetic states, enabling large-scale human studies of epigenetic contributions to ischemic AD risk.

Crucially, epigenetic marks are not static; they can be modified by ongoing experiences across the lifespan, including psychosocial stressors, metabolic states, and inflammatory signals (Feng et al., 2025). Within the circular framework, epigenetic memory serves as a bridge between genetic predisposition and systemic physiological adaptation, translating ischemic signals into lasting biological changes that influence vulnerability to dementia.

### 4.3 Level 3: allostatic load and systemic physiological adaptation

The third level encompasses the cumulative physiological consequences of repeated or chronic adaptation to environmental demands–including ischemic episodes. The concept of allostatic load, introduced by McEwen and Stellar (1993), operationalizes the “wear and tear” on the body resulting from sustained activation of stress-response systems, including the HPA axis, autonomic nervous system, and inflammatory pathways.

Allostatic load is typically measured through composite indices that integrate markers from multiple physiological systems–cardiovascular (e.g., blood pressure, heart rate variability), metabolic (e.g., body mass index, glycated hemoglobin), and inflammatory (e.g., C-reactive protein, interleukin-6) (Guidi et al., 2021). Longitudinal studies have demonstrated that elevated allostatic load is prospectively associated with all-cause dementia and Alzheimer’s disease specifically (Gou et al., 2025; Souza-Talarico et al., 2025). Importantly, allostatic load captures cumulative risk that may not be evident from any single biomarker alone, reflecting the integrated state of multiple physiological systems.

In the ischemic context, allostatic load may be both a cause and a consequence of ischemic injury. Individuals with elevated allostatic load prior to an ischemic event may be more vulnerable to post-ischemic gene dysregulation due to pre-existing systemic inflammation, HPA axis dysregulation, and impaired resilience (Souza-Talarico et al., 2025). Conversely, ischemia itself contributes to allostatic load accumulation through sustained neuroinflammation, oxidative stress, and behavioral changes (Smith et al., 2025).

Smith et al. (2025) showed that allostatic load, as a measure of cumulative physiological stress, is associated with impaired cognitive resilience in older adults. Importantly, they demonstrated that allostatic load effects were independent of amyloid burden, suggesting that systemic physiology may contribute to cognitive decline through pathways distinct from classical neuropathological cascades.

Buller-Peralta et al. (2025) extended these findings to a Chilean older-adults cohort, revealing distinct physiological aging trajectories that were associated with cognitive outcomes, suggesting that allostatic load captures culturally and contextually relevant aspects of physiological aging.

Within the model, Level 3 functions as a critical integrator of signals from higher (Levels 4–5) and lower (Levels 1–2) levels. Chronic psychological stress (Level 4) is associated with allostatic load accumulation, while epigenetic modifications (Level 2) influence how effectively individuals regulate stress responses. Conversely, elevated allostatic load may feedback to induce further epigenetic changes and exacerbate neuroinflammatory processes, establishing vicious cycles that may accelerate cognitive decline.

### 4.4 Level 4: the Psycho-Neuro-Endocrine-Immunological (PNEI) network

The fourth level captures the dynamic interactions among psychological processes, neurological function, endocrine signaling, and immune activity. This PNEI network embodies the bidirectional communication pathways that link mental states, brain function, peripheral physiology, and inflammatory responses (Dantzer et al., 2008; Slavich and Irwin, 2014).

In the ischemic context, the PNEI network is critically involved in mediating the transition from acute injury to chronic neurodegeneration. Dantzer et al. (2008) established the foundational framework for understanding how peripheral inflammation signals the brain, inducing sickness behaviors and cognitive deficits. Their work demonstrated that pro-inflammatory cytokines communicate with the brain through multiple pathways—including vagal afferents, circulating cytokine transport, and endothelial activation—triggering neuroinflammation that can persist long after the peripheral insult resolves.

Slavich and Irwin (2014) advanced this framework through the Social Signal Transduction Theory of Depression, which delineates how psychosocial stressors activate inflammatory responses that contribute to neuropsychiatric disorders. Their model provides a mechanistic bridge between psychological states (Level 4), immune function (Level 4), and brain health.

Feng et al. (2025) synthesized recent evidence on central-peripheral neuroimmune dynamics in psychological stress and depression, revealing bidirectional pathways through which stress alters immune function and immune signals modulate neural activity. They highlighted the role of the blood-brain barrier as a dynamic interface that regulates neuroimmune communication, with implications for dementia pathogenesis.

Within dementia pathogenesis, PNEI dynamics are increasingly recognized as central rather than peripheral. Li et al. (2025) demonstrated that inflammation-mediated regional brain alterations are associated with mild cognitive impairment in knee osteoarthritis, illustrating how peripheral inflammatory conditions can influence central pathology. Similarly, Verduzco Espinoza et al. (2025) showed that microglia-to-neuron signaling links APOE4 and inflammation to enhanced neuronal lipid metabolism and network activity, providing a mechanistic bridge between genetic risk, neuroimmune dynamics, and neural circuit function.

The PNEI network functions as a hub within the circular model, integrating signals from psychological states (e.g., stress, mood), neurological activity (e.g., network oscillations), endocrine status (e.g., cortisol rhythms), and immune function (e.g., cytokine profiles). Perturbations at any point in this network may propagate bidirectionally, potentially amplifying maladaptive states that increase dementia vulnerability.

In the ischemic context, this means that post-ischemic inflammation may not only damage neural tissue directly but may also induce systemic immune changes that perpetuate a pro-inflammatory state, contributing to sustained neurodegeneration.

### 4.5 Level 5: interoceptive-neuronal integration

The fifth level concerns the integration of interoceptive signals—sensory information about the internal state of the body—with neuronal dynamics across large-scale brain networks. Interoception, defined as the sense of the physiological condition of the body (Craig, 2002), provides the foundation for emotional experience, self-awareness, and adaptive regulation of internal states (Barrett and Simmons, 2015; Khalsa et al., 2018).

Recent advances have revealed that interoceptive processing relies on intrinsic neural timescales–the temporal windows over which neurons and neural populations integrate information. Hazelton et al. (2025a) demonstrated that patients with behavioral-variant frontotemporal dementia exhibit altered intrinsic neural timescales of interoceptive processing, with disruptions in allostatic-interoceptive networks that support adaptive regulation. Critically, these alterations were associated with clinical severity and predicted disease progression, suggesting that interoceptive dysfunction is not merely a correlate but potentially a driver of neurodegeneration.

Hazelton et al. (2025b) further showed, through neuroimaging meta-analyses, that interoception, emotion, and social cognition converge across neurodegenerative diseases, including Alzheimer’s disease. This convergence suggests that disturbances in interoceptive-neuronal integration may constitute a transdiagnostic mechanism of cognitive and behavioral decline.

In the ischemic context, interoceptive dysfunction may be both a consequence and a perpetuator of pathology. Ischemic damage to interoceptive networks–particularly the anterior insula, anterior cingulate cortex, and salience network–may impair the brain’s capacity to accurately sense and regulate internal states, leading to maladaptive stable states that manifest clinically as cognitive impairment and behavioral dysregulation (Hazelton et al., 2025a,b).

Within the model, interoceptive-neuronal integration represents the highest-order integrative level within the circular flow of information, where signals from genetic, epigenetic, physiological, and PNEI levels converge to shape conscious experience, self-regulation, and adaptive behavior. When interoceptive-neuronal integration breaks down, the organism loses its capacity to accurately sense, predict, and regulate internal states, leading to maladaptive stable states that manifest clinically as dementia.

## 5 Discussion

### 5.1 Ischemia as a biological “programmer” of Alzheimer’s disease

The evidence reviewed above supports the hypothesis that cerebral ischemia may act as a biological “programmer” that initiates and perpetuates AD-related molecular pathology. Ischemic episodes trigger the sustained overexpression of AD-associated genes–APP, BACE1, PSEN1, PSEN2, MAPT, and apolipoproteins–that persists for years after the initial insult (Pluta et al., 2020, 2025; Pluta, 2022).

This sustained gene dysregulation is not a passive consequence of neuronal damage but rather an active pathological reprogramming maintained through epigenetic modifications, mitochondrial dysfunction, neuroinflammation, and proteostatic failure. Within the hierarchical-circular model, we conceptualize this as a “freezing” of biological memory–a failure of the system to restore adaptive circular information flow after an allostatic challenge (Ruesga Mundo, 2026).

The transdiagnostic nature of this framework is particularly important. The same genes and pathways dysregulated by ischemia are implicated in sporadic AD, suggesting that ischemia may be a common pathway to neurodegeneration. This has significant implications for prevention and therapeutic research: interventions that restore allostatic integrity may be effective across multiple neurodegenerative conditions.

### 5.2 Allostatic integrity as a moderator of ischemic AD risk

The concept of allostatic integrity–the capacity of the organism to maintain adaptive circular information flow across multiple levels–may explain why individuals with similar ischemic injury follow divergent clinical trajectories (Ruesga Mundo, 2026; Smith et al., 2025). When allostatic integrity is preserved, the system may tolerate substantial ischemic damage without clinical expression. When allostatic integrity is compromised, even modest ischemic injury could trigger cascading dysfunction across levels, culminating in dementia.

This hypothesis is supported by studies demonstrating that allostatic load moderates the relationship between neuropathological burden and cognitive decline (Souza-Talarico et al., 2025; Gou et al., 2025). Individuals with high allostatic load show faster progression from mild cognitive impairment to dementia, independent of amyloid and tau status. This suggests that allostatic integrity—the dynamic capacity to resist the cumulative effects of allostatic load—may be a critical determinant of clinical trajectory.

The model generates several discriminative predictions regarding allostatic integrity and ischemic AD:

- Among individuals with similar ischemic injury (e.g., comparable infarct volume or hypoxic-ischemic encephalopathy severity), those with high allostatic integrity will show slower cognitive decline than those with low allostatic integrity.
- Allostatic integrity will moderate the protective effect of cognitive reserve: high reserve may protect against cognitive decline only when allostatic integrity is preserved. When allostatic integrity is low, even high reserve may be insufficient to prevent decline (Ruesga Mundo, 2026).
- Interventions that restore allostatic integrity (e.g., anti-inflammatory agents, stress reduction, metabolic optimization, cognitive training) may attenuate ischemia-induced gene dysregulation and slow disease progression; these remain research hypotheses requiring empirical testing.

### 5.3 Implications for therapeutic research

The hierarchical-circular model suggests that therapeutic strategies for ischemic AD should target the restoration of allostatic integrity across multiple levels rather than focusing on single molecular pathways (Ruesga Mundo, 2026; Livingston et al., 2020).

- Genetic-epigenetic level: Epigenetic modulators (e.g., histone deacetylase inhibitors, DNA methyltransferase inhibitors) may reverse ischemia-induced epigenetic changes and restore normal gene expression (Panitch et al., 2024; Yusupov et al., 2024). These agents are currently under preclinical investigation for various neurological conditions, but their specific application to post-ischemic AD requires systematic study.
- Cellular-mitochondrial level: Antioxidants, mitochondrial enhancers, and agents that promote mitophagy may reduce oxidative stress and restore bioenergetic function (Pluta and Ulamek-Koziol, 2024). While these classes show promise in animal models, clinical translation remains uncertain.
- Synaptic-neurotrophic level: Neurotrophic factors, synaptic enhancers, and cognitive training may promote synaptic plasticity and restore neural connectivity.
- Neuroimmune-proteostatic level: Anti-inflammatory agents, microglial modulators, and proteostasis regulators may reduce neuroinflammation and prevent protein aggregation (Feng et al., 2025; Tyagi and Bartley, 2025). These represent active areas of preclinical and early clinical investigation.
- Behavioral-interoceptive level: Cognitive rehabilitation, physical exercise, and stress reduction may reduce allostatic load and promote adaptive coping (Livingston et al., 2020; Fratiglioni et al., 2020).

It is critical to emphasize that these are presented as research hypotheses derived from the model, not as validated clinical recommendations. Each proposed intervention requires rigorous preclinical and clinical testing specifically in post-ischemic populations before any therapeutic application can be considered. The model’s contribution is to provide a mechanistic rationale for why multidomain approaches might succeed where single-target strategies have often failed.

The FINGER trial (Kivipelto et al., 2018) demonstrated that multidomain interventions (diet, exercise, cognitive training, vascular risk management) can slow cognitive decline in at-risk older adults. The circular model provides a mechanistic rationale for this approach: because levels are coupled through bidirectional feedback loops, interventions targeting multiple levels may disrupt maladaptive circular dynamics more effectively than single-domain approaches. The model further predicts that such interventions may show multiplicative, rather than additive, effects—a testable hypothesis for future trial design.

### 5.4 Toward an allostatic integrity index for ischemic AD

A critical next step is developing validated composite indices of allostatic integrity for use in ischemic AD research. Building on the provisional framework proposed by Ruesga Mundo (2026), we propose the following components as a research-grade operationalization requiring empirical validation:

**Table:** 

Domain	Proposed Markers	Rationale
Adaptive reserve	Heart rate variability (high frequency), awakening cortisol response (normal slope), DHEA/cortisol ratio	Reflects HPA axis and autonomic flexibility
Inflammatory resilience	IL-10/IL-6 ratio, CRP (< 3 mg/L), TNF-α	Captures inflammatory balance and resolution capacity
Multi-system synchrony	Correlation between epigenetic age (Horvath clock) and physiological stress markers	Reflects coherence across levels
Interoceptive accuracy	Heartbeat detection task accuracy, anterior insula functional connectivity	Captures interoceptive-neuronal integration
Circular recovery	Speed of return to baseline (cortisol, HRV, cytokines) after acute stress challenge	Reflects system’s capacity to restore homeostasis

This operationalization requires empirical validation in future studies. The model predicts that composite indices of allostatic integrity will outperform single biomarkers in predicting post-ischemic AD progression (Ruesga Mundo, 2026).

### 5.5 Reconciling clinical paradoxes through a systems lens

The model’s most immediate contribution is its ability to reconcile several long-standing paradoxes that linear models struggle to explain.

Paradox 1: Dissociation between pathology and clinical expression. The observation that individuals with substantial amyloid and tau pathology may remain cognitively intact while others with minimal pathology decline rapidly (Savva et al., 2009) has challenged the amyloid cascade hypothesis for decades. The circular model resolves this paradox by positing that neuropathology is one input among many into a dynamic system. When allostatic integrity is preserved, the system can tolerate substantial pathology without clinical expression. When allostatic integrity is compromised, even modest pathology could trigger cascading dysfunction across levels. Allostatic integrity thus may function as a moderator of pathology’s clinical impact (Ruesga Mundo, 2026).

Paradox 2: The role of early-life stress in late-life dementia. The finding that childhood adversity is associated with dementia risk decades later (Livingston et al., 2020) is difficult to reconcile with models that focus on late-life protein aggregation. The circular model explains this through epigenetic embedding: early-life stress induces lasting epigenetic modifications (Klengel et al., 2013; Yusupov et al., 2024) that calibrate stress reactivity and allostatic load dynamics across the lifespan. These early influences may propagate upward through the system, shaping vulnerability decades before clinical symptoms emerge.

Paradox 3: The failure of single-target therapies. Despite clear evidence that amyloid and tau are involved in Alzheimer’s disease, anti-amyloid therapies have produced only modest clinical benefits (Cummings et al., 2024). The circular model explains this by emphasizing that dementias are systems-level disorders; targeting a single molecular entity while leaving other levels dysregulated is unlikely to restore allostatic integrity. This may explain why combination approaches that target multiple levels–such as the FINGER trial’s multidomain intervention (Kivipelto et al., 2018)–have shown more promising results.

### 5.6 Testable predictions

The hierarchical-circular model generates several falsifiable predictions relevant to the ischemic contribution to AD:

Prediction 1: Composite indices of allostatic integrity will outperform single biomarkers in predicting post-ischemic AD progression. Using existing longitudinal cohorts such as the Alzheimer’s Disease Neuroimaging Initiative (ADNI) or the UK Biobank, researchers can construct composite indices of allostatic integrity that integrate markers from Levels 2 (epigenetic), 3 (allostatic load), 4 (neuroimmune), and 5 (interoceptive). The model predicts that such composite indices will show higher area under the curve (AUC) values in predicting conversion from mild cognitive impairment to dementia compared to amyloid PET status, CSF p-tau181, or any single biomarker alone (Ruesga Mundo, 2026).

Prediction 2: Multidomain interventions targeting more than one system level will have multiplicative, rather than additive, effects on slowing cognitive decline. Randomized controlled trials can be designed to test this prediction by comparing: Group A (single-domain intervention), Group B (single-domain intervention targeting a different level), Group C (combined intervention targeting both levels), and Group D (combined intervention targeting three or more levels). The model predicts that Group C will show effects greater than the sum of Groups A and B (multiplicative interaction), and Group D will show progressively larger multiplicative effects (Ruesga Mundo, 2026).

Prediction 3: Patients with similar ischemic injury but contrasting allostatic integrity will show markedly different trajectories of clinical progression. Among individuals matched for infarct volume or hypoxic-ischemic encephalopathy severity, those with high allostatic integrity will show slower cognitive decline than those with low allostatic integrity. This prediction can be tested using cohorts of stroke survivors or cardiac arrest patients with comprehensive biomarker and cognitive assessment.

Prediction 4: Allostatic integrity moderates the protective effect of cognitive reserve in post-ischemic patients. Among individuals matched for ischemic injury and cognitive reserve proxies (e.g., education, occupational complexity), those with low allostatic integrity will show faster clinical progression than those with high allostatic integrity. Conversely, among individuals matched for allostatic integrity, the protective effect of cognitive reserve will be attenuated when allostatic integrity is low. This crossover pattern is unique to the circular model and differentiates it from reserve frameworks alone (Ruesga Mundo, 2026).

Prediction 5: The genes dysregulated by ischemia (APP, BACE1, PSEN1, PSEN2, MAPT, apolipoproteins) will show coordinated expression changes consistent with a “frozen” biological memory state. This can be tested through transcriptomic analysis of post-ischemic brain tissue, examining whether these genes show persistent coordinated dysregulation that correlates with allostatic load and clinical outcomes (Pluta et al., 2020; Pluta, 2022).

### 5.7 Limitations

Several limitations must be acknowledged. First, much of the evidence for the ischemic contribution to AD comes from animal models, and the translation to human disease requires careful validation (Pluta and Ułamek-Kozioł, 2019; Pluta and Ulamek-Koziol, 2024). We have modified our language throughout to reflect this limitation. Second, the temporal dynamics of post-ischemic gene expression in humans remain incompletely characterized. Third, the causal relationship between ischemia and AD—whether ischemia directly contributes to AD pathology or merely accelerates pre-existing pathology—requires further investigation; we now frame this as a contribution rather than an established etiology. Fourth, the operationalization of allostatic integrity requires empirical validation, and the proposed composite indices must be tested across diverse populations (Ruesga Mundo, 2026). Fifth, the model’s strength—its integration of multiple levels—is also a source of complexity, requiring multimodal data and sophisticated analytical approaches (Serrano-Pozo et al., 2024). Sixth, the model was developed with Alzheimer’s disease as the primary focus, but its principles may extend to other dementias (Hazelton et al., 2025a,b). Future work should test whether allostatic integrity predicts outcomes in vascular dementia, frontotemporal dementia, and Lewy body dementia.

## 6 Conclusion

The evidence reviewed in this article supports a fundamental reconceptualization of the ischemic contribution to Alzheimer’s disease. Cerebral ischemia is not merely a risk factor or a transient insult but rather a biological “programmer” that may initiate and perpetuate AD-associated gene dysregulation across multiple levels of biological organization. However, we emphasize that current evidence does not establish ischemia alone as sufficient to initiate AD as defined by the 2024 NIA-AA biological criteria; rather, ischemia may act as a significant contributor that accelerates or exacerbates AD-related molecular changes in vulnerable individuals.

Within the hierarchical-circular model of biological memory, ischemia may “freeze” biological memory into a pathological configuration, creating self-perpetuating feedback loops of gene dysregulation, mitochondrial dysfunction, synaptic loss, neuroinflammation, and cognitive decline. This framework integrates diverse lines of evidence–from epigenetics to neuroimmunology to clinical neuroscience—into a unified hypothesis that explains the persistent nature of post-ischemic neurodegeneration.

The concept of allostatic integrity emerges as a dynamic systems-level property that may explain why individuals with similar ischemic injury follow divergent clinical trajectories. When allostatic integrity is preserved, the system may tolerate substantial ischemic damage without clinical expression. When allostatic integrity is compromised, even modest ischemia could trigger cascading dysfunction across levels, culminating in dementia.

The implications for therapeutic research are profound. Rather than targeting single molecular pathways, research strategies should aim to restore allostatic integrity across multiple levels. This may require combination approaches that address epigenetic dysregulation, mitochondrial dysfunction, synaptic loss, neuroinflammation, and behavioral factors simultaneously. We stress that these are research hypotheses, not clinical recommendations, and each requires rigorous empirical testing in post-ischemic populations.

In the broader context of AD research, the hierarchical-circular model offers a conceptual roadmap for understanding the ischemic contribution to sporadic AD and for developing biomarker-guided, multidomain research strategies. By reframing AD as a failure of biological memory and allostatic integrity, this framework provides a new direction for research in the field of Alzheimer’s disease and related dementias.

## Data Availability

The original contributions presented in this study are included in this article/supplementary material, further inquiries can be directed to the corresponding author.
